# Synthetic lethal gene pairs: Experimental approaches and predictive models

**DOI:** 10.3389/fgene.2022.961611

**Published:** 2022-12-01

**Authors:** Shan Tang, Birkan Gökbağ, Kunjie Fan, Shuai Shao, Yang Huo, Xue Wu, Lijun Cheng, Lang Li

**Affiliations:** ^1^ College of Pharmacy, The Ohio State University, Columbus, OH, United States; ^2^ Department of Biomedical Informatics, College of Medicine, The Ohio State University, Columbus, OH, United States; ^3^ Indiana University, Bloomington, IN, United States

**Keywords:** synthetic lethality, gene–gene interaction, machine learning (ML), computational biology, predictive model

## Abstract

Synthetic lethality (SL) refers to a genetic interaction in which the simultaneous perturbation of two genes leads to cell or organism death, whereas viability is maintained when only one of the pair is altered. The experimental exploration of these pairs and predictive modeling in computational biology contribute to our understanding of cancer biology and the development of cancer therapies. We extensively reviewed experimental technologies, public data sources, and predictive models in the study of synthetic lethal gene pairs and herein detail biological assumptions, experimental data, statistical models, and computational schemes of various predictive models, speculate regarding their influence on individual sample- and population-based synthetic lethal interactions, discuss the pros and cons of existing SL data and models, and highlight potential research directions in SL discovery.

## 1 Introduction

### 1.1 The concept of synthetic lethality

Synthetic lethality refers to genetic interactions in which the simultaneous perturbation of two genes results in cell or organism death, whereas viability is maintained when only one of the pair loses function. The SL concept was initially developed in model organisms, including fruit flies (Dobzhansky, 1946; Lucchesi, 1968) and yeast (Kaiser and Schekman, 1990; Bender and Pringle, 1991). When crossing fruit flies, early researchers observed that flies harboring concurrent mutations in both the non-allelic *Bar* and *glass* genes died in early stages of development, whereas the presence of mutation in only one of the genes did not affect viability (Sturtevant, 1956; Lucchesi, 1968). Now we know that these two genes encode transcription factors that direct cell processes and that the simultaneous disruption of this encoding function in both genes results in neural defects and death. Early investigators also noted the lethal effect for embryogenesis caused by the simultaneous disruption of homeobox (*HOX*) genes (Lewis, 2000), which were initially discovered in *Drosophila melanogaster* and later found as a family of transcription factors that regulate embryogenesis and morphogenesis. Moreover, Hartwell’s group (Hartwell et al., 1997) proposed extrapolating the synthetic lethal interactions observed in yeast to explore SL-based anticancer therapeutic targets in humans, and in so doing, McManus et al. (2009) demonstrated similar synthetic lethal killing effects in yeast as well as cancer cell lines from the mutation of homologous genes of *RAD54* and *RAD27* ***.***

Eventually, this concept of synthetic lethality was proposed as a basis for the investigation of drug therapies for human diseases. As a form of context-dependent essentiality, the investigation of synthetic lethal genetic interaction has emerged as a powerful approach to the study of cancer-related vulnerabilities. A genetic alteration, such as a defect in a specific tumor suppressor gene (the context), can cause a second gene to become essential for the proliferation of those tumor cells. Thus, in principle, selectively targeting this second SL gene in the presence of the first genetic alteration would be lethal to the tumor cells alone. This SL paradigm has been extensively studied in biomarker discovery, cancer therapeutics, and clinical translation (Sturtevant, 1956; Kaiser and Schekman, 1990; Bender and Pringle, 1991; Lewis, 2000). One salient example is the identification of the synthetic lethal gene pair, *BRCA* and *PARP*, which led to the development of *PARP* inhibitor therapies, e.g., niraparib, for patients with ovarian or breast cancers with *BRCA* mutations (Hartwell et al., 1997).

### 1.2 Is synthetic lethality conserved during clonal evolution or a sample-specific property?

The complex nature of human genes has led to the adoption of simplified model organisms in various studies. The high conservation of many genetic features and pathways between organisms throughout evolution allows the use of less biologically complex model organisms than cell lines, animal models, and humans. Studies of *HOX* genes in fruit flies, as an example of an evolutionarily highly conserved family, have contributed to the understanding of the role of these genes in tumorigenesis and their potential use as therapeutic targets in human cancer (Feltes, 2019; Feng et al., 2021). In particular, the interplay demonstrated between *HOX* genes and DNA repair pathways (Feltes, 2019) has shown the prospect of translation into evolutional studies to identify synthetic lethal gene partners and novel combination treatments for cancer. Boone et al. (2007) has recently generated a global yeast synthetic lethal network that involves 90% of the yeast genome and can possibly be translated across a wide range of cancer cell types.

Advances in new technologies, including RNA inference (RNAi) and clustered regularly interspaced short palindromic repeats (CRISPR), have led to the broader application of SL concepts and subsequent screening efforts in *in vitro* and *in vivo* systems and the acquisition of data revealing new insights regarding the mechanisms of SL. Importantly, studies facilitated by these new techniques suggested that SL was more heterogeneous than homogeneous in cancer. In one instance, using a combinatorial CRISPR technique, Horlbeck’s (Horlbeck et al., 2018) screening of 222,784 gene pairs in K562 and Jurkat leukemia cell lines revealed the SL of 1678 pairs in K562 and 454 pairs among Jurkat lines; the two cell lines shared only 128 (0.057%) of these gene pairs. In a different study facilitated by combinatorial CRISPR, Shen et al. (2017) targeted three cell lines, A549, HELA, and 293T, and found no overlapping synthetic lethal gene pairs among 2628 gene pairs. Although these SL screening studies did not explore the entire genome, they generated new data suggesting that most synthetic lethal gene pairs were cancer cell-specific. This type of specificity can be evident, considering that cell states diverge in the process of clonal evolution during tumorigenesis, and that an evolutionarily conserved SL mechanism can be rare in cancer. Thus, context dependencies might be more evident for regimens based on the general principle of synthetic lethality than those that target single genes (Nijman and Friend, 2013). SL studies based on specific samples or cancer cells are therefore merited for exploring genetic interactions and identifying novel drug combinations to improve cancer treatment.

### 1.3 Limitations of current reviews on synthetic lethality

The general understanding of genetic interaction networks gained from model organisms has wider importance in cancer biology and therapeutics. The identification of evolutionarily conserved genes in yeast, as an illustration, has led to the discovery and characterization of crucial biological phenomena and thereby contributed to the understanding of molecular mechanisms underlying cancer development. Several groups have also reviewed technological advances in the exploration of genetic interactions based on model organisms, especially yeast (Dixon et al., 2009; Adames et al., 2019; Ferreira et al., 2019).

SL research in cancer biology and clinical science has received a great deal of attention. Kaelin (2005) reviewed the SL concept and proposed several chemical and genetic tools (short interfering RNAs, short hairpin RNAs or other interfering RNAs) for perturbing gene functions in cells. Ten years later, O’Neil et al. (2017) further promoted SL screening using genome-editing technologies such as RNAi and CRISPR, and in 2020, Huang’s laboratory reviewed the use of new genome-editing technologies, including combinatorial CRISPR, for the detection of synthetic lethal genes and their application in cancer target discovery (Huang et al., 2020). Though not focused on the prediction of synthetic lethality, in computational biology research, Deng et al. (2019) reviewed the concepts of mutually exclusive genes and genetic interactions and their corresponding computational methods, and Wang et al. (2022a) more recently conducted a much more comprehensive review of SL-related data resources and computational methods.

Still, none of these reviews adequately covered both SL experiments and prediction models, especially with respect to connections between the two investigative methods. Nor did they clarify whether current SL predictive models were based on individual samples or on a population, or whether they provided sufficient detail for the development of predictive SL models. As indicated in Section 1.2, SL is more likely to be sample-specific than population-based or an evolutionary property; so ideally, a predictive SL model should be developed from individual samples or cell lines.

## 2 Experimental approaches

### 2.1 Synthetic lethality experiments

**FIGURE 1:**
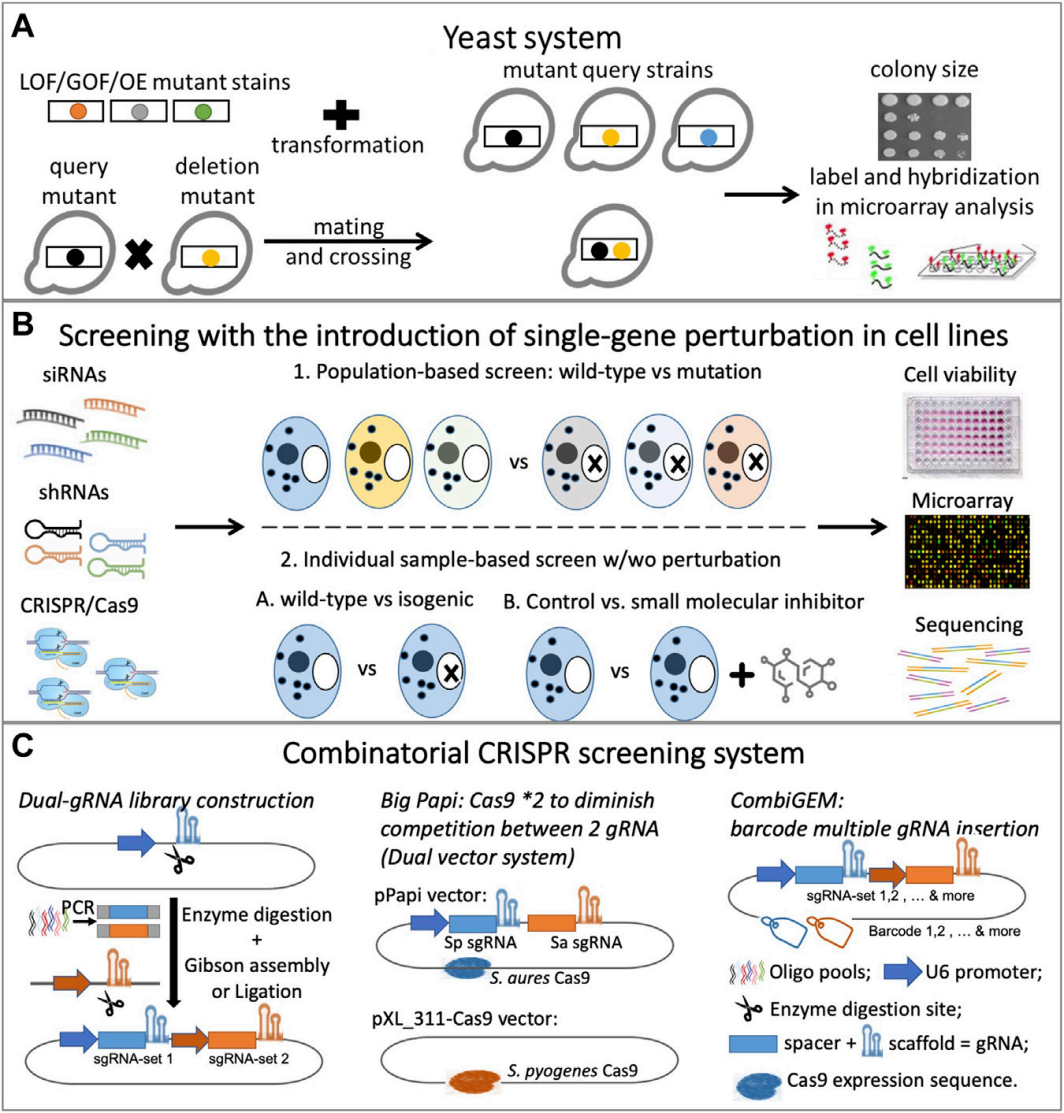
Overview of synthetic lethality experiments: **(A)** Yeast system with double mutant introduced by either transformation or mating; **(B)** Screening with single-gene perturbation introduced by mutation, chemical inhibitor, RNAi or CRISPR; **(C)** Combinatorial CRISPR screening system: dual-gRNA library construction and optimization.

Experimentally, synthetic lethality is determined primarily by identifying gene pairs whose simultaneous disruption causes organism death. Before the discovery of RNAi, SL screens primarily employed chemical compounds or model organisms, such as yeast. RNAi-based gene targeting provided the first opportunity to scale up the screening capacity and systematically identify SL interactions in human cells. More recently, the adaptation of CRISPR and the CRISPR-associated nuclease Cas9 (CRISPR/Cas9) system and the concept of gene essentiality has further facilitated SL screens with higher specificity, efficiency, and flexibility. Figure 1 presents an overview of synthetic lethality experiments.

#### 2.1.1 Synthetic lethality screening using a yeast system

The yeast, *Saccharomyces cerevisiae*, with its stable haploid state, well-annotated genome, and short generation times, has served for over 50 years as a powerful tool in the investigation of gene functions and interactions. In yeast, SL was traditionally discovered by the random mutagenesis of loss-of-function (LoF), gain-of-function (GoF), or overexpression (OE) of mutant query strains followed by a selection regimen, such as drug treatment (Albertini and Zimmermann, 1991; Stevenson et al., 2001). However, yeast-based SL screens are now routinely conducted using systematic screening of arrayed yeast strain collections or arrayed plasmid collections following either of two common methods (Figure 1A). The first approach involves transforming a collection of OE/LoF/GoF mutant strains into a collection of yeast with mutant strains to produce double mutants (Boone et al., 2007); the second involves crossing two sets of mutant yeast strains to obtain haploid double mutants (Segrè et al., 2005; Boone et al., 2007). The crossed mutant strain collections were subjected to synthetic genetic array analysis (SGA) (Tong et al., 2001; Kuzmin et al., 2016), diploid-based synthetic lethality analysis on microarrays (dSLAM) (Pan et al., 2004), and the ‘green monster’ (Suzuki et al., 2011). Recently, Charles Boone led researchers in generating a global SL network with more than 23 million double mutants that crossed 90% of the yeast genome, thereby identifying thousands of SL gene pairs and providing a diagram of the cell’s functional wiring (Costanzo et al., 2016). Based on SGA methodology, they developed trigenic-SGA (τ-SGA) to systematically screen and quantify trigenic interactions in yeast (Kuzmin et al., 2021a; Kuzmin et al., 2021b). The relatively recent introduction of CRISPR to study the yeast system has limited its usage, but Peccoud’s team has described more potential applications for its use in yeast (Adames et al., 2019).

In the yeast system, colony size is typically phenotyped to measure the effects of a single or double mutation on yeast growth/fitness (Baryshnikova et al., 2010), and other measurements, including microarray (Pan et al., 2004) and fluorescence (Suzuki et al., 2011), are also commonly used. Studies of SL in yeast have provided invaluable information regarding fundamental molecular processes that can be used for subsequent screens in higher-level organisms; Nielsen’s research team has summarized the advantages of yeast-based technologies in cancer biology (Ferreira et al., 2019).

#### 2.1.2 Synthetic lethality screening in human cells

RNAi- and CRISPR-based genome-editing technologies have greatly influenced SL screening capabilities. RNAi is a biological process in which an RNA molecule contributes to sequence-specific gene silencing via translational or transcriptional regression, and use of this process provided the first opportunity to knock down (KD) the expression of individual genes and allowed for high-throughput screening in human cells (Laufer et al., 2013). The recent discovery and adaptation of the CRISPR/Cas9 system also brings more flexibility to genetic perturbation. RNAi functions at the post-transcriptional level, but CRISPR/Cas9 has been engineered to introduce functional knock-out (KO) at the gene level. Easily programmable and highly effective, CRISPR-based gene editing has outperformed RNAi. Several research groups have compared RNAi- and CRISPR-based screening technologies (Haussecker, 2016; Housden and Perrimon, 2016; Morgens et al., 2016; Smith et al., 2017).

Conventionally, the SL screens in human cells were categorized based on the techniques employed. For example, Brough’s group (Brough et al., 2011) summarized three methods for identifying SL: 1) applying RNAi screens on cell lines with or without a mutated targeted gene, 2) using RNAi library screens in combination with chemical inhibitors, and 3) chemical library screens. Here, we group the SL screens—chemical inhibitor, RNAi, or CRISPR—based on the level of gene perturbation introduced during the screening. This categorization also lays the foundation for SL calculation (Section 2.2).

SL screenings involving single-gene perturbation compared to cell-line outcomes with or without perturbation of a targeted gene to identify SL partners of that gene. Both population- and individual sample-based screenings apply single-gene perturbation techniques (Figure 1B), but the two approaches differ in the number of cell lines used in the screening. Individual sample-based screenings examine a single cell line, whereas population-based screens utilize multiple cell lines with varied cancer backgrounds. In contrast, SL screenings facilitated by combinatorial gene perturbations involve simultaneous disruptions of two genes within a specified cell line. The SL gene pairs are then identified by determining significant differences between the observed and expected phenotypes. This type of screening is typically based on individual samples.

##### 2.1.2.1 Synthetic lethality screening with the introduction of single-gene perturbation

Within the Cancer Dependency Map portal (DepMap) of the Broad Institute, Project Achilles (depmap portal, 2021) provides a single dataset for population-based SL screening that comprises genome-editing screenings of over one thousand human cell lines, and the projects, DRIVE (Novartis) (McDonald et al., 2017) and SCORE (Sanger Institute) (Behan et al., 2019), provide other genome-editing screening datasets. Using the population-based approach, cell lines for a targeted gene are categorized into those lines either with or without (wild-type, WT) the mutated targeted gene. The SL genes paired with specified target genes are then identified as the genes that are essential among the cell lines with the mutated target gene but not essential among the WT cell lines without the mutation. For example, the synergistic effects between *KRAS* and *STK33* were identified by short hairpin RNA (shRNA) screening between *KRAS*-mutant cell lines (NOMA-1, MDA-MB-231, …) and *KRAS*-WT cell lines (THP-1, MDA-MB-453, …) (Scholl et al., 2009).

In screening individual cell lines, a library of single gene-level perturbations (either RNAi or CRISPR) is introduced into the same cell lines with or without the presence of specified perturbations. Genotype-selective SL can then be identified from the pre-existing perturbation of mutations, and drug-specific SL can be identified by pre-existing perturbations from chemical inhibitors. Compared to the population cell line approach, individual cell line screening allows WT and perturbed cells to share the same genomic background. The genes, *EGFR* (Astsaturov et al., 2010; Pathak et al., 2015), *BRCA* (Lord et al., 2008; Turner et al., 2008), *RAS/KRAS* (Luo et al., 2009; Scholl et al., 2009; Steckel et al., 2012), and *MYC* (Toyoshima et al., 2012) have predominantly been investigated in various screens to identify corresponding genotype-selective synthetic lethal partners. Drug-specific SL can best be illustrated by the discovery of SL between *BRCA2* and *PARP1* inhibitors and their successful application in the clinic (Bryant et al., 2005; Farmer et al., 2005). The CRISPR system makes screening for synthetic lethal drug targets in human cancers feasible at the genome-wide scale, and Surrallés’ research team has summarized the most up-to-date CRISPR screenings to identify genetic interactions (Castells-Roca et al., 2021).

##### 2.1.2.2 Synthetic lethality screening by the introduction of combinatorial gene perturbations

Intuitively, the strategy of utilizing a combinatorial chemical inhibitor or RNA inhibitor (coRNAi) against multiple targets should have been applied to identify SL. Grimm and Kay summarized the development of the coRNAi strategy and its potential application in a clinical setting (Grimm and Kay, 2007), but few studies have employed this highly labor-intensive methodology. Furthermore, the limited number of inhibitors available for various targets challenges the scaling up of SL identification using only combinatorial chemical inhibitors even more. The recent development of CRISPR, specifically combinatorial CRISPR screening, allows for the systematic detection of SL genetic interactions by massive parallel pairwise gene disturbance. The simultaneous incorporation of dual guide RNA (gRNA) pairs into the expression vector permits double perturbation in the screen and has become the basic lead for the combinatorial CRISPR technique (Figure 1C).

Vidigal and Ventura (2015) first established a one-step method of cloning specific gRNA-pairs into any CRISPR-expression vector starting from pools of short oligonucleotides, and the construction of a double-KO gRNA library has undergone continuous modification and optimization. Wong’s laboratory developed combinatorial genetics *en masse* (CombiGEM) for the extensible assembly of barcoded high-order combinatorial screens (Wong et al., 2016; Han et al., 2017; Zhou et al., 2020); Najm et al. (2018) developed Big Papi, a dual-Cas9 system to diminish competition for Cas9 protein between two gRNAs; and Boettcher et al. (2018) combined two orthogonal Cas9 proteins allowing for quantification of LoF and GoF phenotypes in the same screen. Among currently published combinatorial CRISPR screens, extensive effort has been exerted to reveal the SL between paralogues, such as *FAM50A/FAM50B* (Thompson et al., 2021), *DUSP4/DUSP6* (Ito et al., 2021), and *CDK4/CDK6* (Parrish et al., 2021). However, though the ability to screen gene combinations has grown, library size and cell culture still constrain the capacity for combinatorial CRISPR screening. For example, screening a library of 5000 gRNA pairs with standard conditions of 100 coverages and multiplicity of infection (MOI) of 0.3, the initiation of screening for each replicate sample will require at least 1.6 million cells. The largest study comprised 1,044,484 gRNA pairs targeting 111,392 gene pairs in K562 and Jurkat cells (Horlbeck et al., 2018), and more recently, Diehl et al. (2021) implemented a multiplexing method they termed 3Cs to generate combinatorial CRISPR libraries with low distribution skews, allowing the lowering of cell coverage and total cell numbers in one screen.

### 2.2 Experimental approaches to the calculation of synthetic lethality

SL is determined under different experimental settings by identifying gene pairs whose simultaneous disruption causes organism death, and its calculation varies depending on the experimental design. The consideration of gene essentiality, a founding and dynamic concept of genetics, has recently brought new perspectives to SL identification. A gene is judged essential if it is required for the reproductive success of an organism under specific conditions. As mentioned in section 2.1, essentialities can be variously quantified by measuring yeast colony size, cell viability, or gRNA abundance in surviving populations. SL between two genes occurs when neither gene is essential, but perturbation of both genes compromises proliferation or fitness.

In RNAi-based screening, changes in cell viability are primarily phenotyped. The most common cell assays include CellTiter, AlarmBlue, MTT, and Luminescent ATP (Stoddart, 2011; Adan et al., 2016). Microarrays are also used in RNAi-based screens to measure the representation of shRNA/small interfering RNA (siRNA), especially for a relatively large library. Sequencing of CRISPR screens is typically required to quantify the inserted gRNA read counts.

#### 2.2.1 Calculation of synthetic lethality in single-gene-perturbation screening

The SL partners of a targeted gene (gene of interest) are identified by comparing gene essentialities between two groups of cell lines, either with or without perturbation of second-query genes (query strains in yeast). In population-based screening, these two groups are cells with target gene mutations and wild-type cells without the mutations. In cell-specific screening, the two groups are wild-type cells and cells with a mutated target gene or under certain perturbations using chemical inhibitors, RNAi, or CRISPR. SL calculation involves comparison of a gene’s essentiality between two groups, mainly by difference (Bommi-Reddy et al., 2008), fold-change or abundance ratio (Lord et al., 2008; Boettcher et al., 2014), Z-score (Turner et al., 2008; Martin et al., 2009; Steckel et al., 2012; Toyoshima et al., 2012; Shen et al., 2015), or *t*-test (Luo et al., 2009) of gRNA counts. Scoring by methods such as RNAi gene enrichment ranking (RIGER) (Luo et al., 2008), gene activity ranking profile (GARP) (Marcotte et al., 2012a), and observation of redundant siRNA activity (RSA) (König et al., 2007) is also commonly used for SL calculation.

#### 2.2.2 Calculation of synthetic lethality in double-perturbation experiments

The methods for calculating SL from data generated by double-perturbation experiments, such as double-mutant yeast, combinatorial RNAi, or CRISPR screening, can be placed into two categories. The first approach introduces growth phenotype and calculates the deviation of the observed growth phenotype from the expected growth phenotype for a specified gRNA-gRNA pair. The growth phenotype is measured by the change in frequency of the initial and surviving populations for single-gRNA (gRNA-safe gRNA pair) or gRNA-gRNA (Wong et al., 2016; Han et al., 2017; Horlbeck et al., 2018; Najm et al., 2018; Parrish et al., 2021), and the expected growth phenotype is then calculated by summation (Han et al., 2017; Parrish et al., 2021; Thompson et al., 2021) or quadratic fitting (Horlbeck et al., 2018) of the growth phenotypes of two single-gRNAs. These two sgRNAs will be identified as synthetic lethal partners if the observed phenotype of the paired gRNAs is significantly lower than the expected value, suggesting a notable shift in gene essentiality with the presence of the two gRNAs. The gene–gene interaction can be calculated from the average (Horlbeck et al., 2018; Parrish et al., 2021) or ranking (Han et al., 2017; Thompson et al., 2021) for corresponding sgRNA pairs.

A different approach models the combination effect of double perturbation as a two-way analysis of variance (ANOVA) with interaction. Two perturbations are considered synthetic lethal if they lead to significant decline in individual fitness compared with their combined additive effect (Shen et al., 2017; Zhao et al., 2018). Individual fitness can be measured either as cell viability, the change in frequency of inserted gRNA fragment in the surviving cells over time (Shen et al., 2017; Zhao et al., 2018), or, as discussed in 2.1, by colony size in yeast experiments (Baryshnikova et al., 2010; Costanzo et al., 2016). A variational Bayesian approach (Ito et al., 2021) or Dunnett’s test (Zhou et al., 2020) can also be used to calculate SL in combinatorial CRISPR screening.

## 3 Synthetic lethality data

Table 1 categorizes SL data into four groups: 1) curated databases that utilize information from multiple sources; 2) library-based repositories of data from genomic screening and multi-omics profiling; 3) collections of data from SL screenings with the introduction of single perturbation (discussed in Section 2.1.2.1), and 4) based on combinatorial perturbation (discussed in Section 2.1.2.2).

**TABLE 1:** Curated databases and library-based screening data.

Data sources	Data description
Curated databases	
DepMap depmap portal (2021)	Dependency Map: loss-of-function (LoF) screens by Project Achilles of the Broad Institute, cell-line multi-omics by the Cancer Cell Line Encyclopedia (CCLE), and drug-sensitivity profiles by PriSM. https://depmap.org/portal/
DRIVE McDonald et al. (2017)	Large-scale short hairpin RNA (shRNA) screens on 7837 genes across 398 cell lines with CCLE features. https://oncologynibr.shinyapps.io/drive/
SGD Skrzypek and Nash (2015)	*Saccharomyces* genome database: yeast genome sequences, functional annotations, expression profiles, gene–gene interactions; includes over 10,000 synthetic lethal interactions for more than 6600 genes. https://www.yeastgenome.org/
SynLethDB 2.0 Wang et al. (2022b)	Synthetic lethal pairs and non-synthetic lethal pairs for human, fly, worm, mouse, and yeast, including the gene identification numbers of the National Center for Biotechnology Information (NCBI), PubMed ID of the study, the source that classified the gene-pair interaction, and the statistical score between 0 (low) to 1 (high). Recently updated from version 1.0, the databases now house a total of 50,868 interactions for 13,707 genes. http://synlethdb.sist.shanghaitech.edu.cn/v 2/
SLKG Zhang et al. (2021)	Synthetic lethality knowledge graph: 19,987 synthetic lethal pairs and 3039 synthetic dosage lethal (SDL) pairs with SL score between 0 (low) and 1 (high) calculated from 11 external databases (DRIVE, DepMap and SynLethDB,…). https://www.slkg.net/
Library-based genomic screenings and multi-omics profiling data	
TCGA Tomczak et al. (2015)	The Cancer Genome Atlas pan-cancer database: 85,415 patient samples (33 major cancer types) with microarrays, DNA sequencing, tissue imaging, methylation (Micheel et al., 2018) (137). https://www.cancer.gov/about-nci/organization/ccg/research/structural-genomics/tcga
CCLE Barretina et al. (2012); Broad Institute (2005)	Cancer cell line encyclopedia: gene expression (1389 cell lines), mutation (1755 cell lines), and copy number (1750 cell lines). https://sites.broadinstitute.org/ccle/
GTEx Lonsdale (2013)	Genotype-tissue expression project: gene expression for different tissue types (17,382 samples over 948 donors in 54 non-diseased tissues) using both bulk-cell and single-cell gene profiles. https://gtexportal.org/home/
Specialized datasets	Curated microarray database (CuMiDa) (Feltes et al., 2019): 78 human microarray datasets curated from the Gene Expression Omnibus (GEO) https://sbcb.inf.ufrgs.br/cumidaBARRA:CuRDa (Feltes et al., 2021): 17 human RNA-seq datasets curated from the GEO https://sbcb.inf.ufrgs.br/barracurdaCancerSCEM (Zeng et al., 2022): 208 single-cell RNA-sequencing samples from 28 studies, covering 20 human cancer types https://ngdc.cncb.ac.cn/cancerscem/

Data Sources: synthetic lethality screening based on single-gene perturbation				
Data sources	Library size	Target	Technology	Cell line/s
Astsaturov et al. (2010)	Targeting 638 genes with two siRNAs for each gene	*EGFR*	siRNA screen with chemical inhibitors	A431
Bommi-Reddy et al. (2008)	100 shRNAs targeting **88** kinases	Von Hippel-Lindau (*VHL*)	shRNA screen	786-O (WT), RCC4 (*VHL*−/−)
Lord et al. (2008)	Human DNA repair siRNA set V1.0 (siRNA library containing **230** DNA repair genes).	*PARP* and DNA repair genes	siRNA screen with *PARPi*	CAL51
Turner et al. (2008)	siRNA library targeting 779 human protein kinase and kinase-related genes.	Human protein kinase and kinase-related genes	siRNA screen with *PARP*i	CAL51
Luo et al. (2009)	74,905 retroviral shRNAs targeting 32,293 unique human transcripts.	*KRAS*	GW shRNA screen	DLD-1 *Ras* WT, DLD-1 *Ras* Mut
Martin et al. (2009)	**1200** drugs and drug-like molecules	*MSH2*	Gene mutation with chemical inhibitor screening	Hec59 (*MSH2* deficient), Hec59 + chr2 (*MSH2* proficient, WT)
Marcotte et al. (2012a)	78,432 shRNAs targeting 16,056 Ref-seq genes (O'Leary et al., 2016).	Ref-seq genes	shRNA screen	29 breast, 28 pancreatic, 15 ovarian cancer cell lines
Steckel et al. (2012)	7000 siRNA pools targeting the druggable human genome (∼7400 genes).	*KRAS*	shRNA screen	HCT116 (*KRAS* mut), HKE-3 (WT)
Toyoshima et al. (2012)	siRNAs targeting 3300 druggable genes and 200 microRNAs.	*MYC*	siRNA screen	HFF-MYC (overexpression), HFF-PBabe (control)
Vizeacoumar et al. (2013)	78,432 unique shRNAs targeting 16,056 human genes.	*PTTG1*, *BLM*, *MUS81*, *PTEN*, *KRAS*	siRNA screen	HCT116 with derived phenotypes of PTTG1−/−, BLM−/−, MUS81−/−, PTEN−/−, KRAS^+/−^
Boettcher et al. (2014)	Pooled shRNAs targeting 10,000 genes, together with siRNAs targeting fumarate hydratase (FH).	Fumarate hydratase	shRNA and siRNA screen	HEK293T, UOK262
Shen et al. (2015)	Targeting 112 known tumor-suppressor genes.	*CHEK1/2*	siRNA screen with chemical inhibitor AZD7762	HeLA (treated with siRNAs together with AZD7762 versus DMSO)
Pathak et al. (2015)	1276 siRNAs targeting 638 genes	A network centered on *EGFR*, *HER2*, *BCAR1*, *NEDD9*, and *EFS*	siRNA screen with chemical inhibitor dasatinib	A1847

Synthetic lethality screening based on combinatorial perturbations			
Combinatorial perturbation studies	Library size	Synthetic lethality biology	Cell lines (disease versus normal)
Laufer et al. (2013)	51,680 siRNA combinations that come from 323 epigenetic regulator genes	Epigenetic regulation genes	HCT116
Wang et al. (2014)	6032 siRNA pairs of **1508** gene pairs	Genes with frequent alterations in breast cancer	MCF10A
Wong et al. (2016)	50 genes, 23,409 gRNA pairs	Epigenetics regulation	OVCAR8
Han et al. (2017)	207 genes, 21,321 gRNA pairs	Non-essential drug targets	K562
Shen et al. (2017)	73 genes, 141,912 gRNA pairs	Drug targets	HeLa, 293T, A549
Horlbeck et al. (2018)	472 genes, 111,392 gene pairs	Non-essential genes	K562, Jurkat
Zhao et al. (2018)	51 genes,11,475 gRNA pairs	Metabolic network	HeLa, A549
Ito et al. (2021)	3284 genes, 5065 paralog pairs	Paralog families	11 cancer cell lines
Parrish et al. (2021)	2060 gene pairs	Paralog families	PC9, HeLa
Diehl et al. (2021)	160 genes, 12,736 gene pairs	Autophagy pathway	HEK293T, RPE1
Thompson et al. (2021)	1191 gene pairs	Paralog families	A375, MeWo, RPE1

When SL scores from different studies are combined in either a curated database approach, such as that using SynLethDB (Guo et al., 2016) or 2.0 (Wang et al., 2022b), or a collection of generated big data, such as that employing a synthetic lethality knowledge graph (SLKG) (Zhang et al., 2021), each SL pair receives a new computed score from 0 to 1 that reflects the strength of their interaction, with higher scores indicating stronger interactions. In general, because the new SL computation is dependent on data-driven approaches rather than the depiction of SL interactions from the original studies, the new scoring schemes vary greatly across different approaches, deviating notably from original sources, and making the SL scores less comparable.

## 4 Predictive models of synthetic lethality

Predictive models are categorized into two general types based on whether the input data are population-based or individual sample-based. Population-based models identify SL gene pairs from a population of samples, whereas individual sample-based methods predict SL pairs by considering features of the sample of interest. All rule-based statistical inference models and network models are population-based, and machine-learning models can be either population- or individual sample-based. Multi-omics features, covering gene expression, somatic mutation, somatic copy number alteration (SCNA), and protein–protein interactions (PPI) are frequently adopted to improve modeling performance.

**FIGURE 2:**
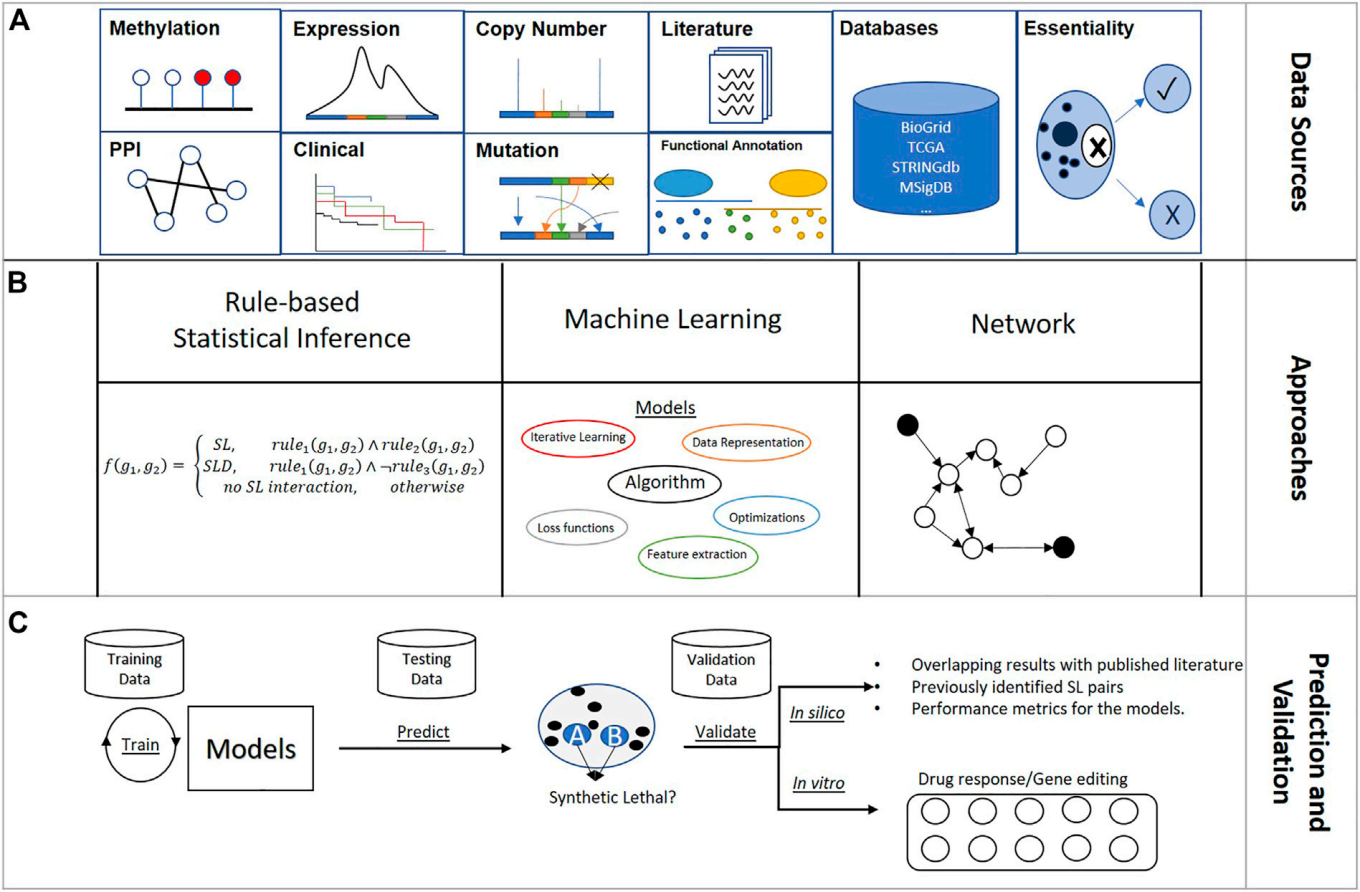
Overview of synthetic lethality predictive model design: **(A)** Main sources used to engineer gene features for SL identification; **(B)** Three main model architectures for creating SL prediction model; **(C)** General pipeline of SL predictive modeling.

In this section, the review of each SL predictive model focuses on the input data, modeling process, and validation. Figure 2 summarizes approaches to the design of SL predictive models.

### 4.1 Population-based models

#### 4.1.1 Rule-based statistical inference models

Largely built on the assumptions derived from the SL concept, rule-based inferences use statistical tests to infer synthetic lethal gene pairs at the population level from single-gene-based genome-editing screenings and multi-omics profiling data. Table 2 presents the biological assumptions, sources of input data, and statistical tests.

**TABLE 2:** Biological assumptions, sources of input data, and network statistics for predictive models.

		Synthetic lethal interactions, biological assumptions.	Input data sources						Statistical tests
			Gene expression	SCNA	Somatic mutation	Phylogenetics	Clinical patient data	Short hairpin RNA	
Statistical inference	DAISY Jerby-Arnon et al. (2014)	Gene pairs that overlap across all assumptions.	☑	☑	☑			☑	Wilcoxon rank sum, followed by Bonferroni correction for multiple hypothesis testing; gene co-expressions were calculated using Spearman correlation
		1. Survival of the fittest (SoF): Synthetic lethal pairs are co-inactivated for cell death.							
		2. Death upon single gene knockdown when another gene is inactive is synthetic lethality.							
		3. Synthetic lethal pairs are co-expressed.							
	Srihari et al. Mutual Exclusivity Model (Srihari et al., 2015)	Gene pairs that are frequently altered in a mutually exclusive manner are defined as synthetic lethal.	☑	☑					The statistical significance value was obtained by subtracting SL score obtained by hypergeometric test from 1: $p_{val}=1-SL_{hypergeometric}$
	ISLE Lee et al. (2018)	Gene pairs that exhibit the following characteristics:	☑	☑	☑	☑	☑		Statistical significance tests used for the respective assumptions:
		1. Gene pairs are rarely co-inactivated compared to their individual inactivation frequencies.							1. Hypergeometric test
		2. Gene pairs yield better patient survival through their co-inactivation, reducing tumor fitness when co-inactive.							2. Likelihood ratio test
		3. Gene pairs tend to co-evolve and thus have high phylogenetic similarity.							3. No statistical test at this step
									Afterward, Wilcoxon rank sum was used to compare identified SL pairs with drug target response
	ASTER Liany et al. (2020a)	Gene pair (Genes A and B) that passes the following tests:	☑	☑	☑	☑			Wilcoxon rank sum, followed by Fisher’s method for combining significance *p*-values. False discovery rates were determined using the Benjamini–Hochberg method
		1. For tissue-specific samples with high Gene A copy number, the expression level of Gene A is significantly higher than that of non-cancerous samples of the same tissue type.							
		2. For tissue-specific samples with high Gene A copy number, but low Gene B copy number, expression level of Gene B is significantly lower than that of non-cancerous samples of the same tissue type.							
		3. Expression levels of Gene A in Test 1 is significantly higher than those of Gene B in Test 2.			☑				
	SLIdR Srivatsa et al. (2019)	Synthetic lethal pairs consist of a significantly mutated gene and its interacting genes that yield cell death upon co-occurrence of their aberrations.		☑	☑			☑	Custom, rank-based statistical test was used where the *p*-value was obtained from the lower-tail probability
	MiSL Sinha et al. (2017)	The mutations of synthetic lethal pairs are amplified more frequently and are deleted less frequently while in concordance with their gene expression profiles.	☑	☑	☑				Fisher’s exact test for evaluating gene-pair behavior dependence, followed by two-tailed unpaired Student’s t-test
Network-based models	VIPER Alvarez et al. (2016)	A probabilistic framework where tissue-specific gene-expression data are used to identify regulator-target interactions following the activation or repression of a regulator.	☑						Analytic rank-based enrichment analysis (aREA) statistical analysis is used to discern differential gene activity
	OptiCon (Hu et al., 2019)	Using gene expression profiles in a regulatory network, optimal control nodes (OCNs) are identified such that they exert maximal control over deregulated pathways, but minimal control over unaffected pathways for a given disease. For SL tasks, OCNs point to potential synthetic lethal pairs	☑	☑					Wilcoxon rank test and one-sided Kolmogorov-Smirnov test

The data-mining synthetic-lethality-identification pipeline (DAISY) algorithm (Jerby-Arnon et al., 2014) is the first rule-based statistical-inference approach to identify and evaluate SL gene pairs from The Cancer Genome Atlas (TCGA) of the National Cancer Institute (NCI) and the National Human Genome Research Institute (NHGRI), the Cancer Cell Line Encyclopedia (CCLE), and DepMap. The first rule is called the genomic survival of fitness. Specifically, for a SL gene pair, A and B, it is assumed that if A is inactive (based on SCNA, gene expression, and somatic mutation), B will not be deleted or have a high copy number. The Wilcoxon rank test is used to compare the Gene B copy number between clinical samples with the active or inactive Gene A. The Wilcoxon rank test is also used with the second rule, to test whether cancer cells with inactive Gene A (based on SCNA and gene expression) are more likely than those with active A to have essential Gene B (based on shRNA screenings). For the third rule, a positive Spearman’s correlation coefficient is applied to investigate the co-expression of synthetic gene pairs A and B. Several published RNAi experiments in human cell lines have demonstrated the utility of DAISY.

Srihari et al. (2015) proposed a statistical method based on the concept of mutual exclusivity that assumed the likelihood that combinations of genes that exhibit mutual exclusivity in genetic events are synthetic lethal. They considered six key DNA-damage response (DDR) genes that are frequently altered across four cancer types (breast, prostate, ovarian, and uterine). The assessment of SCNA and gene expression of TCGA-identified genes that were altered in a mutually exclusive manner was based on a hypergeometric test with these six DDR genes as synthetic lethal partners. This model was validated against GARP essentiality scores from *in vitro* studies (Marcotte et al., 2012b; Vizeacoumar et al., 2013).

The identification of clinically relevant synthetic lethality (ISLE) (Lee et al., 2018) utilizes three criteria, employing a large initial pool of laboratory-identified candidate SL pairs determined either by double-knockout screens or guilt-by-association using large-scale single gene knock-out experiments as inputs. First, gene expression and SCNA data were used to identify candidate gene pairs whose co-inactivation was less frequent than expected as calculated using a hypergeometric test of their individual inactivation frequencies. Second, a gene pair was selected if its co-inactivation led to better predicted patient survival in TCGA samples according to the Cox proportional hazards model. In the last step, ISLE considered the tendency of functionally interacting genes to co-evolve and calculated phylogenetic similarity across 86 species in a tree of life structure using non-negative matrix factorization to select SL pairs comprising genes with strong phylogenetic similarity. Initial candidate gene pairs satisfying all three conditions were validated on other datasets (Barretina et al., 2012; Costello et al., 2014; Gao et al., 2015; Menden et al., 2019), and prediction performance was tested by phenotypic drug response screens *in vivo*.

Analysis of synthetic lethality by comparison with tissue-specific disease-free genomic and transcriptomic data (ASTER) (Liany et al., 2020a) predicted SL gene pairs for both cancerous tissues using SCNA from TCGA and disease-free tissues using gene expression data from the genotype-tissue expression project, GTEx, of the NHGRI. The main consideration of ASTER is whether cancer samples are tissue-specific when the gene-pair, A-B, exhibits a pattern of mutual exclusivity. Using disease-free tissues from GTEx as reference, in Test 1, ASTER selected disease samples with a high copy number in Gene A, and then compared the expression of Gene A in these samples to that in the reference GTEx samples. In Test 2, a subset of disease samples with a low copy number for Gene A and high copy number for Gene B was selected, and the expression of Gene B in this sample subset was then compared to that of the reference GTEx samples. Test 3, the final test, assessed whether the expression levels of Gene A were significantly higher than those of Gene B between two diseases. The Wilcoxon rank sum test was performed for each of the three tests and followed by Fisher’s method to combine the *p*-values. ASTER utilizes fewer datasets and has a simpler framework for hypothesis testing than DAISY and ISLE and outperformed those methods.

Synthetic lethal identification in R (SLIdR) (Srivatsa et al., 2019) is a statistical framework for identifying SL pairs from large-scale perturbation screens, including essentiality profiles from Project DRIVE with corresponding mutation and SCNA from CCLE. RSA was used to compute the gene-level essentiality score of each cell line (König et al., 2007) with a cutoff value of ˗3 in more than 50% of the cell lines. Driver genes for each cancer type were defined using the Broad Institute’s mutation significance file (MutSig MAF) from TCGA (Lawrence et al., 2014), focusing on genes demonstrating significant mutations in cancer samples; cell lines were sorted into mutated or WT groups based on the mutation status of the driver genes. SLIdR aims to identify synthetic lethal interactions between a driver gene and another perturbed gene based on a statistical test ranking essentiality scores across all perturbed genes for each mutated versus WT cell line. SLIdR identifies synthetic lethal gene pairs based on the assumption that a mutation in the driver gene in combination with knockdown of the perturbed gene yields lower essentiality scores compared to scores in the WT group. A one-sided statistical test based on the Irwin-Hall distribution is used to determine statistical significance. Of the potential synthetic lethal pairs identified by SLIdR, only one of the top synthetic lethal pairs, *AXIN1* and *URI1*, was validated *in vitro* in this paper.

The algorithm for mining synthetic lethals (MiSL) (Sinha et al., 2017) extracts human pan-cancer data for 12 specific types of cancer from the TCGA dataset to identify mutation-specific synthetic lethal partners. Its underlying assumption is that a mutated gene’s synthetic lethal partners will be amplified more frequently or deleted less frequently in samples that harbor the mutation and concordant changes in expression across multiple cancers. MiSL aims to identify partners of gene B that have more copies in the presence of mutated Gene A based on Boolean implications of either preferred amplification in the presence of the mutation or deletion only in the absence of the mutation as determined using Fisher’s exact test and maximum likelihood estimation. Two filtering steps are applied afterward to increase accuracy. First, candidate genes serving merely as passengers are excluded. An example of a passenger is a deletion in Gene A that is not differentially down-regulated in samples with deletions in A compared to the rest of samples. Second, only genes that are differentially overexpressed in the presence of the mutation versus the WT based on a *t*-test are retained to form the final candidate SL partners. MiSL’s successful identification of SL interaction between mutation in *IDH1* and *ACACA* in leukemia was validated by gene targeting and patient-derived xenografts.

#### 4.1.2 Network models

Network models select single genes or gene combinations as potential drug targets based on the network’s topology. Though the criteria for selecting gene combinations are technically irrelevant to the concept of synthetic lethality, many gene pairs selected from network models are potentially SLs. Table 2 shows biological assumptions, input data sources, and network statistics for network models.

Virtual inference of protein activity by enriched regulon analysis (VIPER) (Alvarez et al., 2016) evaluates the functional relevance of genetic interactions in regulatory proteins based on gene-expression data from TCGA. VIPER requires accurate cellular networks that are highly dependent on tumor context (Margolin et al., 2006). Based on a probabilistic framework that includes target status (activated or repressed, with or without overlapping) and statistical confidence, VIPER applies an optimized rank-based analysis to compute the enrichment of a protein’s regulon in differentially expressed genes. VIPER is frequently used to infer aberrant protein activity from gene expression, and the correlation between regulator and target genes generated from the probabilistic framework in VIPER provides valuable information for context-specific gene–gene interactions and has potential use in SL prediction.

The optimal control node (OptiCon) algorithm (Hu et al., 2019) is a network controllability-based method to identify synergistic key regulators as candidate targets for combination therapy. OptiCon constructs a gene regulatory network from three pathway databases−the reactome pathway knowledge base (Jassal et al., 2020), the Kyoto Encyclopedia of Genes and Genomes (KEGG) (Kanehisa and Goto, 2000), and the NCI-nature pathway interaction database (Krupa et al., 2007)−and obtains gene-expression data of tumor tissues with matched normal tissues from TCGA for calculating gene deregulation scores. OptiCon first assesses a disease-perturbed gene regulatory network (DRN) to identify a set of optimal control nodes (OCNs) in a specified disease that controls the transition of the network between any two conditions. The identification of OCNs is formulated as a combinatorial optimization problem and is solved through a ‘greedy search’ algorithm. OptiCon then identifies synergistic OCN pairs by defining a synergy score that captures both enrichment of recurrently mutated genes (mutation score) and density of crosstalk between pathways (crosstalk score) controlled by a pair of OCNs. The synergistic pairs of OCNs predicted by OptiCon are supported by synthetic lethal interactions from the SynLethDB and the study by Shen’s group (Shen et al., 2017). The top predictions were validated experimentally by CRISPR screening (Han et al., 2017).

#### 4.1.3 Supervised machine-learning models

Supervised machine-learning models learn associations between input features and known SL data to predict novel SL gene pairs using multi-omics data. Table 3 shows how population features and omics features were generated for these machine-learning models.

**TABLE 3:** Population and omics features for machine-learning predictive models.

		Population features			Omics features				
		PPI	Functional annotation	Knowledge graph	Expression	SCNA	Essentiality	Mutual exclusivity	Synthetic lethality network
Population-based	Mashup Cho et al. (2016)	☑							
	CMF Liany et al. (2020b)	☑	☑		☑	☑	☑	☑	☑
	SL^2^MF Liu et al. (2020)		☑						☑
	GRSMF Huang et al. (2019)		☑						☑
	ESML Lu et al. (2015)				☑	☑			
	DDGCN Cai et al. (2020)								☑
	GCATSL Long et al. (2021)	☑	☑						☑
	KG4SL Wang et al. (2021)			☑					
Individual sample-based	MNDT Wong et al. (2004)	☑							☑
	MNMC Pandey et al. (2010)	☑	☑						☑
	DiscoverSL Das et al. (2019)		☑		☑			☑	
	EXP2SL Wan et al. (2020)				☑				

The Mashup algorithm (Cho et al., 2016) involves the topological integration of multiple network types through graphic representation. For SL prediction, Mashup uses the STRING network for protein interactions (Szklarczyk et al., 2021), the Cancer Genome Project for drug-response profiles in cancer cell lines (Garnett et al., 2012), and the gene ontology (GO) (Ashburner et al., 2000) and Munich Information Center for Protein Sequences (MIPS) (Mewes et al., 2002) databases for functional annotation. A random walk with restart is employed to calculate the diffusion and connectivity of each data node within an individual network (Tong et al., 2006). During their integration, calculated gene features are minimized across networks to represent the topology of all networks. Afterward, the generated features along with graphic representation of the data networks are used in machine learning to predict the synthetic lethal interactions specified by Jerby-Arnon et al. (2014). The interactions are defined by the mean and absolute difference between the calculated feature representations across gene pairs and fitted by a support vector machine (SVM) using a programming library (LIBSVM) (Chang and Lin, 2011). The model’s prediction efficacy was validated by data on fifty cancer drugs with single-gene targets in over 639 cell lines obtained from the Cancer Genome Project (CGP).

Collective matrix factorization (CMF) (Liany et al., 2020b) is an unsupervised method that utilizes low rank factorization on design matrix inputs. The datasets used in CMF are represented in the matrix and include protein complex co-memberships from the comprehensive resource of mammalian protein complexes (CORUM) (Giurgiu et al., 2019), human PPI from the human integrated protein–protein interaction reference (Hippie) (Alanis-Lobato et al., 2017), co-expression profiles from the search tool for the retrieval of interacting genes/proteins database (StringDB) (Szklarczyk et al., 2021), and pathway co-membership scores calculated from Broad Institute’s molecular signatures database (MSigDB) (Subramanian et al., 2005). These datasets were factorized together to target the SL interactions data from the research groups of Laufer et al. (2013), Vizeacoumar et al. (2013), Shen et al. (2017), and Zhao et al. (2018), and from the SynLethDB database. Each input was integrated through similarly annotated rows and columns, and the CMF methods were implemented in three ways: by CMF (low-rank), gCMF (group-sparse CMF using group-sparse prior on columns), and dCMF (deep-learning CMF utilizing multiple auto-encoders for dimensionality reduction). The gCMF method performed the best for tasks inferring synthetic lethal interaction using principal component analysis and graphic features from DAISY. CMF was not validated further; the only reported validation was on the held-out datasets from five datasets used for training.

Synthetic lethality to logistic matrix factorization (SL^2^MF) (Liu et al., 2020) uses logistic matrix factorization to obtain latent protein factors for the prediction of SL pairs. The model’s design adopts a similarity-based drug-target interaction model named BLM-NII (-neighbor-based interaction-profile inferring) (Mei et al., 2013). SL^2^MF uses GO semantic similarity (Ashburner et al., 2000) and PPI data from the human protein reference database (HPRD) (Keshava Prasad et al., 2009) to bolster the model’s predictions in the networks’ topologies. The representative latent gene factors were used in a logistic function to predict SL pairs. The SL data were obtained from SynLethDB and used in 5-fold cross-validation. The SL pairs used as positive training samples exclude the pairs predicted from DAISY and high-scoring SynLethDB pairs. The model’s performance achieved an AUC of 0.85 and an AUPRC of 0.24. The model was validated *in silico* and by comparison with DAISY in overlapping SL predictions within the SynLethDB.

The graph-regularized self-representative matrix factorization (GRSMF) (Huang et al., 2019) model represents a matrix by a linear combination of its rows and columns using SL interactions as the input matrix. In the process, the model is bolstered by graph regularization with GO semantic similarity (Ashburner et al., 2000) and uses a majorization-minimization objective function (Yang and Oja, 2011) in its training The model is applied to SL data from SynLethDB with 5-fold cross-validation and its performance is compared with BLM-NII (Mei et al., 2013), SL^2^MF (Liu et al., 2020), and SMF (GRSMF without graph regularization support). AUC scores demonstrated that GRSMF (0.92) and SMF (0.89) outperformed both SL^2^MF (0.85) and BLM-NII (0.74). The model was validated *in silico.*

The ensemble-based machine-learning model (ESML) (Lu et al., 2015) uses multiple classifiers and multi-omics datasets, including RNA sequencing data generated by the Broad Institute’s firehose suite of tools and pipelines (Tomczak et al., 2015) and SCNA data from the cBioPortal for cancer genomics (Gao et al., 2013), to define gene-pair interaction features, namely homozygous co-loss, heterozygous co-loss, mixed co-loss, co-underexpression, and expression up-down signals. The co-loss signals are derived from gene deletion profiles in SCNA data, whereas co-expression profiles are computed from the RNA-seq data. The model consists of seven different classifiers: the adaptive boosting (AdaBoost) algorithm (Freund and Schapire, 1997), the J48 algorithm (Salzberg, 1993), JRip, a Java-based implementation of the RIPPER algorithm (Cohen, 1995), the Logit function (Lu et al., 2015), the LogitBoost boosting algorithm (Friedman et al., 2000), partial decision trees (PART) (Frank and Witten, 1998), and the random forest algorithm (Breiman, 2001). The same gene-pair interactions were fed into each of the classifiers, and the outcomes showing greatest agreement across classifiers were chosen. The framework is then applied to the synthetic lethal pairs from Laufer’s (Laufer et al., 2013) and Vizeacoumar’s groups (Vizeacoumar et al., 2013) to generate population-based and genome-wide-scale SL interactions. Under a probability threshold of at least 0.81, the model achieved a precision score of 0.67 and recall score of 0.10.

The dual-dropout graph convolutional network (DDGCN) (Cai et al., 2020) is the first graph neural network (GNN) model to predict SL gene pairs. DDGCN proposes a novel dual-dropout mechanism to solve the problem of overfitting associated with the sparsity of SL. Known SL gene pairs are used to construct a synthetic lethal interaction network in which each gene is a node and SL interactions form edges, which allows the prediction of novel SL to be cast as a link prediction task to complete missing edges in the interaction network. The dual-dropout consists of a coarse-grained node dropout that randomly drops some gene nodes during each training iteration, and a fine-grained edge dropout that randomly removes some edges for further fine-tuning. DDGCN has been theoretically justified and validated utilizing the SynLethDB database, with a predicted AUC of 0.85 and AUCR of 0.90.

The graph contextualized attention network to synthetic lethality (GCATSL) (Long et al., 2021) is another GNN-based model that incorporates various biological data sources utilizing graph attention network (GAT) architecture. Compared to a basic GNN model, GAT can effectively distinguish and preserve differences among neighbors by assigning different weights. In GCATSL, three feature graphs were constructed using as input features, biological processes (BPs) and cellular components (CCs) from GO as well as the PPI network from the biological general repository for interaction datasets (BioGRID) (Stark et al., 2006), and a dual-attention mechanism (node- and feature-level attention) that is designed to learn node representations from multiple feature graphs. Specifically, node-level attention was used with GAT to learn preliminary representations for each input feature graph, and feature-level attention was then implemented to integrate these three feature graphs and generate the final representation for each gene node. Prediction performance was validated on the SynLethDB database, with prediction AUC of 0.94 and AUCR of 0.95.

The knowledge graph for synthetic lethality (KG4SL) (Wang et al., 2021) is a GNN-based method that incorporates a knowledge graph (KG) into the prediction of SL. The authors highlighted that existing methods often regarded each SL pair as an independent sample and failed to consider the underlying biological mechanisms; whereas some shared biological factors might latently imply dependency among SL pairs. In contrast, KG4SL considers knowledge graphs involving biological processes, diseases, and compounds. Given the heterogeneous input graph, KG4SL utilizes an attention mechanism to handle the passing of messages of different types of nodes and edges. The inner product between the representations of the gene pair is regarded as the probability of being SL. KG4SL has shown excellent performance in the SynLethDB database, yielding an AUC of 0.95 and AUCR of 0.96.

#### 4.2 Individual sample-based models

These individual sample-based models are all supervised approaches. Table 3 shows how their input features are defined.

The multiple network decision tree (MNDT) model (Wong et al., 2004) utilizes a decision tree classifier to predict SL interactions. Prior to training on the decision trees, the gene pairs are given manually curated features to depict their genetic interaction networks. Data for the networks were obtained from various sources, including MIPS (Mewes et al., 2002) for functional relations and Goldberg and Roth’s physical interaction network (Goldberg and Roth, 2003). A total of 123 gene-pair characteristics, comprising common upstream regulators, gene co-occurrence, and chromosomal distances between genes, were compiled in the genetic network. Interactions of these gene-pairs were then extended to a third gene, designated 2hop that interacts with the other two. For instance, if gene C has a physical interaction with Gene A in one network and synthetically lethal interaction with Gene B in another, then the A-B gene pair is assigned a 2hop-physical-SL characteristic. According to the curated features, the gene pairs were fed into the decision tree to decide which leaf node the input should land on and the location of the node containing the SL classification prediction. Then, the features were trained on SGA-analyzed data from Tong et al. (Tong et al., 2001) and fitted to SL data obtained from an early version of Tong et al. (2004) for validation.

The multi-network and multi-classifier (MNMC) (Pandey et al., 2010) model was extended from the MNDT (Wong et al., 2004) model utilizing multiple classification techniques, including k-nearest neighbors, neural network, random forest, and SVM in addition to decision trees. Individual-network and overlaid-network features, including gene expression, protein–protein interaction, transcription factor binding, and functional annotation profiles from GO and KEGG, were generated from the yeast dataset to predict synthetic lethal interactions. A total of 152 individual network features were identified, 62 of which hinted at stronger connections, such as physically interacting genes within the PPI network. Additionally, 90 overlaid features were generated via 2hop interactions across the networks as described in MNDT. This allowed the model to capture repeated and similar interactions across different network types and to create an integrated representation input dataset. The Kolmogorov-Smirnov test was applied to determine the top features against distinguishing synthetic lethal and non-synthetic-lethal gene pairs, and the model was then trained using synthetic lethal interactions from the *Saccharomyces* genome database (Cherry et al., 1997), as described by Wong et al. (2004). MNMC was validated *in silico* within the training datasets.

The DiscoverSL (Das et al., 2019) model uses clinical and multi-omics data from TCGA (Tomczak et al., 2015) and data from the MSigDB pathway annotation database (Subramanian et al., 2005) to predict synthetic lethal interactions in the SynLethDB. Feature sets of differentially expressed genes, expression correlations, mutual exclusivity, and pathway information were calculated, and a random-forest classifier created with these four features was trained on SL interactions. After the model was trained on SynLethDB, the derived patient-specific SL interactions were validated *in silico* by visualizing shRNA essentiality screens, SCNA targetability, cell-line drug sensitivity data, and Kaplan-Meier survival curves against different gene expression profiles. This model was further validated *in silico* on cell lines from the genomics of drug sensitivity in cancer (GDSC) database (Yang et al., 2013). Other studies, such as that of Origanti and associates regarding *CHEK1* and *p21* (Origanti et al., 2013), confirmed some of the predicted SL interactions.

Expression to synthetic lethality prediction, EXP2SL (Wan et al., 2020), is a machine-learning network based on a semi-supervised neural network. EXP2SL was used to extract gene-expression profiles from the L1000 project of the library of integrated network-based cellular signatures (LINCS) of the National Institutes of Health’s Common Fund (Subramanian et al., 2017), apply a multi-layer fully connected neural network to individually encode the profiles for each input gene pair, and then concatenate the encoded representations to make the final prediction. Because synthetic lethal labels for an individual sample are limited, EXP2SL designs a semi-supervised Bayesian personalized ranking (BPR) loss into the objective function to incorporate a large amount of unlabeled data. Testing of the model on the combinatorial CRISPR SL datasets in three different cell lines (Shen et al., 2017; Najm et al., 2018; Zhao et al., 2018) demonstrated its competitive prediction ability.

## 5 Discussion

### 5.1 Synthetic lethal predictive models are not comparable

Each SL predictive model has its own unique pros and cons, and the models are not comparable. Rule-based statistical-inference approaches predict SL gene pairs based on assumptions derived from the definition of SL and do not require training under experimentally validated SL data. They are therefore routinely applied to multi-omics data collected from clinical samples to allow evaluation of the clinical significance of SL gene pairs through analysis of their association with clinical outcomes. Network-based approaches also do not require training on SL data. They have been applied primarily for the discovery of combinational targets. Population-based supervised machine-learning SL predictions are not specific to individual samples, and sample-specific SL prediction models are only trained and designed for individual samples. The unique assumptions, training data, and purposes of each of these four types of model preclude comparisons between their performances. Variation among published methods and results, input population features, and sample omics features for each model suggest that direct comparisons even within a model are not necessarily feasible. The real challenge to comparing performance among predictive models is that the various published studies do not sufficiently report implementation details, including both programming codes and model tuning parameters, thereby limiting or preventing reproducibility and comparison. We expect that future SL research studies will focus on comparisons within each model type, assessing common input features, training, and validation datasets.

### 5.2 The intrinsic limitation of population-based synthetic lethal models developed from machine-learning algorithms

Most population-based SL predictive models were developed from machine-learning algorithms using SynlethDB 1.0, a biased and outdated database of SL gene pairs. In fact, neither the SynLethDB 1.0 or 2.0 database includes eight of the ten SL screening studies, and the two SL screening studies (Han et al., 2017; Shen et al., 2017) they do include incorporate only 1075 of 20,990 (Shen et al., 2017) and 152 of 2630 (Han et al., 2017) original SL gene pairs. Why and how remaining data points were excluded is unclear.

Furthermore, even if the SynlethDB integrated all published SL datasets, questions remain regarding the methods of training and constructing the population-based SL models and how SL predictions were predicted. A gene pair that is positive in any screening experiment is labeled positive. However, as discussed in section 1.2, overlapping synthetic lethal gene pairs are rare across cell lines or different screenings. In addition, population-based models typically average population features and omics features from a sample set that does not necessarily correspond to any individual sample. So, a predicted SL gene pair is interpreted as SL in one or multiple cells and can be interpreted to reflect neither common SL among all cells nor sample-specific SL. This significantly limits our understanding of why SL occurs in some cells but not others. Moreover, it is unclear whether a gene pair labeled as negative SL is truly negative or if it has simply not been examined. Each new study’s generation of new data will significantly alter both positive and negative labeling of data and a model’s predictions of synthetic lethality.

We suggest that it would be preferable if SL predictive models were built for individual samples and that they were sample-specific. Successfully developed, these models could predict sample-specific SL gene pairs, and common pairs among multiple samples could be identified thereafter.

### 5.3 Disconnection between synthetic lethal experiments and predictive models

Synthetic lethal experiments and predictive models are complementary technologies, but they have not been implemented together. Experiments typically preselect gene-pair inputs based on the study’s objectives. The experiment data yield a confidence score for gene interaction and a cutoff for either statistically or biologically significant SL, whereas predictive models predict SL for any gene pair with confidence reflected in a probability score of 0-1. The combined use of SL experiments and predictive models would be ideal, with each facilitating the outcomes of the other. We wonder, for example, if predictive models might aid the choice of a set of genes or gene pairs for designing experiments that will improve the chances of discovering SL gene pairs, and how we could choose the most appropriate predictive model, a statistical-inference or machine-learning model, for example, that will aid experimental design. These are interesting, important, and currently unanswered questions.

### 5.4 Which synthetic lethal experiment deserves more attention in the development of SL predictive models?

Our review of synthetic lethal experiments focused on two major schemes, one relying on single gene-perturbed screening between two sets of cell lines (perturbation of specific versus wild-type genes) and the second relying on double-perturbation screening. The first scheme scans all combinations between the target and other genes and requires no computational model to predict SL. However, the second combinatorial screen is seriously limited by the number of genes because the number of their combinations increases exponentially. Therefore, the development of an SL predictive model for double-perturbation experiments would be highly valuable in the selection of gene pairs.

### 5.5 What are the opportunities in synthetic lethality predictions?

Many uncharted territories remain for the prediction of synthetic lethality. SL data are usually very limited in or absent from a cancer cell line when we begin its exploration for combination target discoveries, and even when we know a set of synthetic lethal genes in our targeted cell line, the gene set is usually limited. We can utilize rule-based statistics and network- and population-based approaches to aid our selection of the first candidate gene–gene pairs as inputs for synthetic lethal experiments, but research is still needed to determine the best strategy for selecting that initial gene set. It will be interesting to determine which of those methods can help improve the development and performance of individual cell-specific SL predictive models. Finally, traditional experiments usually focus on validating true synthetic lethal gene pairs, but if our goal is to build up a more powerful predictive model, informative training samples from experiments should include both true and negative synthetic lethal gene pairs. To the best of our knowledge, this aspect has not been studied.
